# Pre-existing chronic interstitial pneumonia is a poor prognostic factor of Goodpasture’s syndrome: a case report and review of the literature

**DOI:** 10.1186/s13256-017-1273-8

**Published:** 2017-04-13

**Authors:** Hiroki Tashiro, Koichiro Takahashi, Yuki Ikeda, Saori Uchiumi, Makoto Fukuda, Miyazono Motoaki, Shinya Kimura, Naoko Sueoka-Aragane

**Affiliations:** 1 Division of Hematology, Respiratory Medicine and Oncology, Department of Internal Medicine, Faculty of Medicine, Saga University, 5-1-1 Nabeshima, Saga, 849-8501 Japan; 2 Division of Nephrology, Department of Internal Medicine, Faculty of Medicine, Saga University, Saga, Japan

**Keywords:** Goodpasture’s syndrome, Interstitial pneumonia, Anti-glomerular basement membrane antibody, Myeloperoxidase anti-neutrophil cytoplasmic antibody

## Abstract

**Background:**

Goodpasture’s syndrome is a rare disease that is characterized by rapidly progressive glomerulonephritis and diffuse alveolar hemorrhage.

**Case presentation:**

A 71-year-old Japanese man who had chronic interstitial pneumonia was diagnosed as having Goodpasture’s syndrome. Both anti-glomerular basement membrane antibody and myeloperoxidase anti-neutrophil cytoplasmic antibody were increased. Despite intensive treatments, including mechanical ventilation, he died from respiratory failure. Pathological findings at autopsy showed rapidly progressive glomerulonephritis in his kidneys, diffuse alveolar hemorrhage, hyaline membranes, and fibroblastic foci in his lungs. The cause of death was diagnosed as respiratory failure as a result of diffuse alveolar damage induced by a combination of diffuse alveolar hemorrhage and exacerbation of interstitial pneumonia.

**Conclusions:**

We report a case of Goodpasture’s syndrome complicated with pre-existing chronic interstitial pneumonia and positive myeloperoxidase anti-neutrophil cytoplasmic antibody. We reviewed six similar cases reported in the literature and concluded that Goodpasture’s syndrome with pre-existing interstitial pneumonia and myeloperoxidase anti-neutrophil cytoplasmic antibody is related to a poor prognosis.

## Background

Goodpasture’s syndrome (GPS) is a rare autoimmune disease that is related to anti-glomerular basement membrane (anti-GBM) antibodies [1]. Clinical manifestations are characterized by rapidly progressive glomerulonephritis (RPGN) and diffuse alveolar hemorrhage (DAH) [2]. The incidence of GPS is estimated to be one case per million per year [3]. The pathogenesis of GPS is thought to be related to circulating anti-GBM antibodies inducing RPGN and DAH; however, the mechanism of anti-GBM antibody production is not fully understood [4]. Although treatments with plasmapheresis, corticosteroids, and immunosuppressive agents have improved the prognosis, some patients still die as the disease progresses [5]. Poor prognostic factors related to GPS have been reported to be positive myeloperoxidase anti-neutrophil cytoplasmic antibody (MPO-ANCA) and elevated serum creatinine levels; however, others have not been clearly elucidated [6, 7].

Here we report a rare case of GPS complicated with pre-existing chronic interstitial pneumonia. We reviewed six similar cases reported previously and estimated that GPS with pre-existing interstitial pneumonia and positive MPO-ANCA are related to poor clinical outcomes.

## Case presentation

**Fig. 1:**
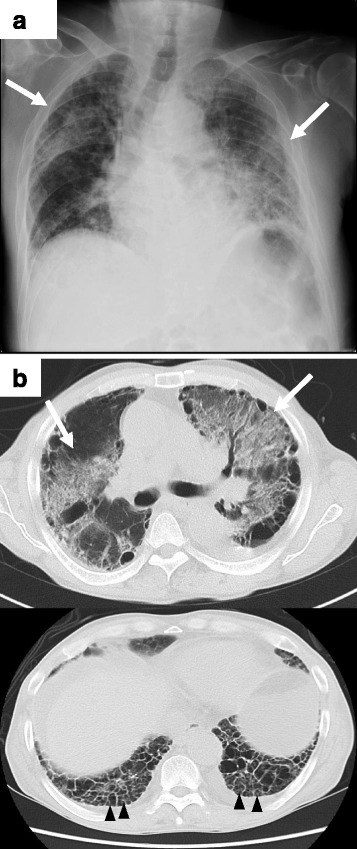
Findings of chest radiograph and computed tomography at admission. **a** *Arrows* show chest radiograph findings of pulmonary infiltrates and ground-glass shadows in both lung fields. **b** *Arrows* show chest computed tomography findings of pulmonary infiltrates with ground-glass shadows. *Arrowheads* show honeycomb shadows in both lung bases

A 71-year-old Japanese man presented to our hospital with complaints of dyspnea, oliguria, and general fatigue. He had baseline intellectual disability and a 50-pack year history of tobacco smoking. He had been presumptively diagnosed with interstitial pneumonia related to tobacco smoking 7 years prior to admission, but a definitive diagnosis had not been made, and annual chest radiographs had shown no progression of interstitial pneumonia. On examination, his respiratory rate was 20 breaths/minute and respiratory crackles were heard in both lungs. He had edema in his extremities but did not have peripheral neuropathy. Echocardiography showed normal cardiac function. A chest radiograph showed diffuse pulmonary infiltrates and ground-glass shadows in both lung fields (Fig. 1a). Chest computed tomography (CT) also showed pulmonary infiltrates with ground-glass shadows and honeycomb shadows in both lung bases, suggesting a usual interstitial pneumonia (UIP) pattern (Fig. 1b). His laboratory findings on admission revealed the following: white blood cell count 13.9 × 10^3^/μL with 90.4% neutrophils; hemoglobin 9.3 g/dL; blood urea nitrogen 80.8 mg/dL; creatinine 11.42 mg/dL; uric acid 8.9 mg/dL; lactate dehydrogenase 290 IU/L; sodium 115 mEq/L; potassium 5.7 mEq/L; chloride 85 mEq/L; calcium 7.4 mg/dL; C-reactive protein 10.24 mg/dL; and KL-6 931 IU/mL. His serum anti-GBM antibody was increased at 151 IU/mL (normal <3.0) and anti-MPO-ANCA was also increased at 9.5 IU/mL (normal <3.5). His platelet count, coagulation profile, and other biochemical tests were within normal range. Urine studies showed proteinuria and microscopic hematuria. Arterial blood gas analysis with supplemental oxygen at 5 L/minute was as follows: partial pressure of oxygen (pO_2_) 74.2 torr; partial pressure of carbon dioxide (pCO_2_) 24.9 torr; pH 7.275; and bicarbonate (HCO_3_) 11.2 mEq/L. He did not undergo a renal biopsy because of his unstable respiratory condition. He was diagnosed as having RPGN caused by GPS. He was initially treated with corticosteroids (prednisolone 1.0 mg/kg per day) and mechanical ventilation. Subsequently, he was treated with plasma exchange every other day. Despite the intensive treatments, his oxygenation worsened. He developed bloody sputum on day 4, and pulmonary infiltrates on his chest radiograph did not improve. Although pulse corticosteroid therapy (methylprednisolone 1000 mg/kg per day) was started on day 4, his oxygenation did not improve. On day 7, he died from respiratory failure. Consent from his family was obtained, and we performed an autopsy.

**Fig. 2:**
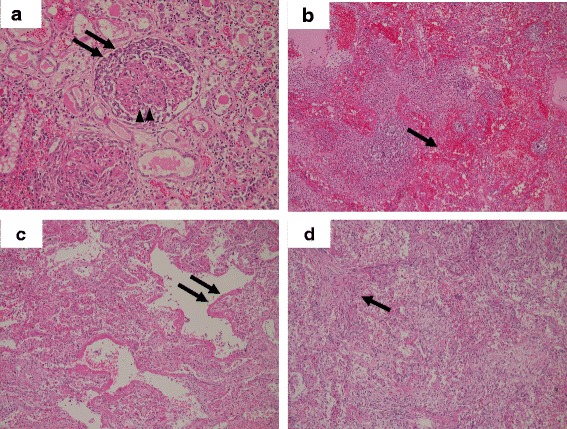
Pathological findings at autopsy. **a** *Arrows* show pathological findings in the kidney of crescentic glomerulonephritis. *Arrowheads* show fibrinous necrosis and infiltration of inflammatory cells (hematoxylin and eosin, ×400). **b** *Arrow* shows pathological findings of the lung showing diffuse alveolar hemorrhage. **c** *Arrows* shows hyaline membrane in the alveolar wall. **d** *Arrow* shows fibrotic hyperplasia of the alveolar walls and fibroblastic foci (hematoxylin and eosin, ×400)

Pathological findings at autopsy showed crescentic glomerulonephritis accompanied by fibrinous necrosis and an infiltration of inflammatory cells in the kidney (Fig. 2a). Lung pathology showed hemosiderin-laden macrophages in the alveolar spaces, fibrinoid necrosis of the alveolar and capillary walls, infiltration of neutrophils in the alveolar walls, and formation of hyaline membranes (Fig. 2b, c). Moreover, fibrotic hyperplasia of the alveolar walls and fibroblastic foci were seen in the lung bases (Fig. 2d). We diagnosed the cause of death as respiratory failure due to diffuse alveolar damage induced by alveolar hemorrhage and exacerbation of chronic interstitial pneumonia.

## Discussion

GPS is a lung-specific and kidney-specific autoimmune disease that is related to anti-GBM antibodies [8]. Clinical manifestations include acute renal failure caused by RPGN and acute respiratory failure caused by DAH [9]. GPS complicated with pre-existing chronic interstitial pneumonia, as seen in our case, is certainly rare.

To the best of our knowledge, six similar cases complicated with pre-existing interstitial pneumonia have been reported [10–12]. We reviewed the six cases along with the present case (Table 1). Six of the seven patients (85.7%) were men, and the duration from diagnosis of interstitial pneumonia to the onset of GPS ranged from 2 months to 9 years. All of these patients were treated with corticosteroids and hemodialysis. In addition, four were treated with plasma exchange, one with cyclophosphamide, and three required mechanical ventilation. The 1-year survival rate of GPS is reported to be between 75% and 90% [1]. Furthermore, that of GPS with positive MPO-ANCA is reported to be 76% [13]. Surprisingly, the 1-year survival rate of our reviewed cases of GPS with pre-existing interstitial pneumonia and positive MPO-ANCA was 29%, and the cause of death in those cases was respiratory failure. DAH has been reported as a trigger that exacerbates chronic interstitial pneumonia [14, 15]. In the present case, lung pathological findings showed diffuse alveolar damage that may have been induced by a combination of DAH and exacerbation of interstitial pneumonia. These findings suggest that exacerbation of pre-existing interstitial pneumonia is a poor prognostic factor for GPS and is related to critical respiratory failure in the acute phase.

**Table 1:** Characteristics of patients with Goodpasture’s syndrome complicated with pre-existing interstitial pneumonia

	Age	Gender (M/F)	Smoking (pack year)	MPO-ANCA	Type of interstitial pneumonia	Duration from onset of IP to GPS	CGN	DAH	Treatments	Survival duration	Cause of death	Reference
1	78	M	50	+	UIP	8 years	+	+	CS, PE, HD, MV	3 months	Respiratory failure	[10]
2	64	M	60	+	UIP	2 months	+	+	CS, HD	3 months	Respiratory failure	[10]
3	72	M	45	+	UIP	N.A.	+	+	CS, PE, HD	6 years	Leukemia	[10]
4	42	F	Never	+	UIP	N.A.	+	+	CS, HD	Alive	N.A.	[10]
5	55	M	30	+	NSIP	9 years	+	+	CS, CY, PE, HD, MV	1 months	Respiratory failure	[11]
6	78	M	102	+	UIP	4 years	+	+	HD, CS	6 months	Respiratory failure	[12]
7	71	M	50	+	UIP	7 years	+	+	CS, PE, HD, MV	7 days	Respiratory failure	present case

*CGN* crescentic glomerulonephritis, *CS* corticosteroid, *CY* cyclophosphamide, *DAH* diffuse alveolar hemorrhage, *F* female, *GPS* Goodpasture’s syndrome, *HD* hemodialysis, *IP* interstitial pneumonia, *M* male, *MPO-ANCA* myeloperoxidase anti-neutrophil cytoplasmic antibody, *MV* mechanical ventilation, *N.A*. not available, *NSIP* non-specific interstitial pneumonia, *PE* plasma exchange, *UIP* usual interstitial pneumonia

The pathogenesis of GPS is not fully understood. The target GBM antigen molecule was subsequently identified as the noncollagenous-1 (NC1) domain of the α3 chain of collagen IV [16]. In addition, environmental factors are also thought to increase the risk of the disease, for instance, respiratory infection by influenza virus, exposure to hydrocarbon fumes or metallic dust, and tobacco smoking [2].

Of interest, almost all of the seven cases we reviewed were positive for serum MPO-ANCA and six of the seven patients had a severe tobacco smoking habit. Pre-existing chronic interstitial pneumonia might be a trigger for the production of MPO-ANCA because MPO-ANCA is known to be positive prior to the development of microscopic polyangiitis (MPA) [17]. In addition, MPO-ANCA-positive interstitial pneumonia and cigarette smoking might be a factor associated with the induction of positive anti-GBM antibody. Moreover, RPGN and positive anti-GBM antibody is reported to be a poor prognostic factor in patients with MPA [18, 19]. According to these findings, we propose that MPO-ANCA-positive interstitial pneumonia is the related lung manifestation of GPS, not just a coexisting disease, and both MPO-ANCA and pre-existing interstitial pneumonia are related to poor clinical outcomes for patients with GPS.

## Conclusions

We report a case of GPS complicated with pre-existing chronic interstitial pneumonia and positive MPO-ANCA. The cause of death was diagnosed as respiratory failure as a result of diffuse alveolar damage induced by a combination of DAH and exacerbation of interstitial pneumonia. We conclude that pre-existing interstitial pneumonia and a positive MPO-ANCA are important prognostic factors in the clinical outcomes of patients with GPS, and chronic interstitial pneumonia with MPO-ANCA may be related to the pathogenesis of GPS.

## Abbreviations

CT: Computed tomography
DAH: Diffuse alveolar hemorrhage
GBM: Glomerular basement membrane
GPS: Goodpasture’s syndrome
HCO_3_: Bicarbonate
MPA: Microscopic polyangiitis
MPO-ANCA: Myeloperoxidase anti-neutrophil cytoplasmic antibody
NC1: Noncollagenous-1
pCO_2_: Partial pressure of carbon dioxide
pO_2_: Partial pressure of oxygen
RPGN: Rapidly progressive glomerulonephritis
UIP: Usual interstitial pneumonia
